# Paraneoplastic cerebellar degeneration and dermatomyositis as first manifestations of underlying breast malignancy: a report of two cases and a brief review of the subject

**DOI:** 10.1186/s40792-015-0063-z

**Published:** 2015-07-16

**Authors:** Ying Ru Yvonne Ng, Chunyin Derek Ho, Weng Leong Victor Ng, Su-Ming Tan

**Affiliations:** Department of General Surgery, Changi General Hospital, 2 Simei Street, Singapore, 529 889 Singapore; Department of Laboratory Medicine, Changi General Hospital, Singapore, Singapore

**Keywords:** Breast cancer, Paraneoplastic, Cerebellar degeneration, Dermatomyositis

## Abstract

Paraneoplastic syndromes are rare first manifestations of breast cancer. In this report, we present two cases of a 58-year-old woman and a 69-year-old woman presenting with acute symptoms of paraneoplastic cerebellar degeneration (PCD) and dermatomyositis, respectively, as the first sign of breast malignancy. The patient diagnosed with PCD presented initially with ataxia, was serum anti-Yo antibody negative, and subsequently investigated to have poorly differentiated intraductal breast carcinoma. Cerebellar symptoms regressed following breast cancer surgery and chemotherapy, highlighting the better neurological prognosis associated with anti-Yo antibody negative PCD. The rarity of these presentations highlights the necessity to include an occult malignancy in the differential diagnosis when attending to such patients.

## Background

Paraneoplastic syndromes are hypothesized to be triggered by an abnormal autoimmune system response to an underlying malignancy or by humoral factors expressed by tumour cells [1]. While breast malignancy commonly presents as a breast lump or a suspicious radiological finding, 1–3 % have non-metastatic-related paraneoplastic manifestations [2]. Several studies have documented the association between breast cancer and dermatomyositis [3], whereas the association between paraneoplastic cerebellar degeneration (PCD) and breast malignancy is less known with only a handful of cases reported. However, few of such cases have been cited in Asia, and to our knowledge, this is the first report for both paraneoplastic presentations in Southeast Asia. We present two women who first showed cerebellar signs and skin rash that were subsequently attributed to underlying breast cancer.

## Case presentation

### Case presentation 1

**Fig. 1:**
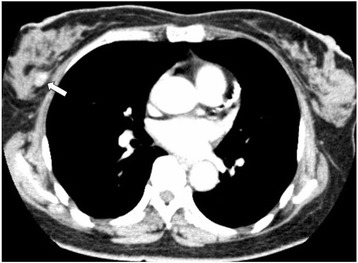
Computed tomography of the chest showing the right breast nodule with irregular margins

A 58-year-old woman with no past medical history or intake of chronic medications experienced progressively worsening vertiginous giddiness and unsteadiness for 1 week, associated with noticeably impaired coordination and speech slurring for 2 days. Neurological examination found gaze-evoked rotatory nystagmus, diplopia, bilateral dysmetria and dysdiadochokinesia, dysarthria and gait ataxia. To diagnose the cerebellar disorder, biochemical, cerebrospinal fluid and radiological tests were performed to rule out infective causes, metabolic causes including hypothyroidism and vitamin B_12_ deficiency, autoimmune causes including celiac disease and glutamate decarboxylase autoantibodies, neurodegenerative disease including Miller Fisher syndrome, and primary or metastatic cerebellar lesion. The only significant biochemical result was a positive antinuclear antibody level. Magnetic resonance imaging (MRI) of the brain for a stroke or cerebellar disorder was negative. Suspicion of paraneoplastic cerebellar presentation was raised, and she was evaluated for a primary lesion. Cerebrospinal fluid (CSF) analysis showed lymphocytes with increased protein oligoclonal bands indicative of intrathecal immunoglobulin (Ig) G synthesis. CSF and serum anti-neuronal antibodies (anti-Yo) were however negative. High-dose intravenous Ig was commenced with no improvement. Computed tomography (CT) scan of the chest, abdomen and pelvis detected a right breast ten o’clock enhancing nodule with irregular margins associated with enlarged axillary and subpectoral nodes (Fig. 1). Breast imaging confirmed multicentric breast lesions with axillary adenopathy compatible with malignancy and nodal metastasis. Breast biopsy established a grade 3 invasive ductal carcinoma (IDC) with oestrogen, progesterone and HER-2 receptor statuses negative.

Pathological staging returned as T_1B_N_1_M_0_ (stage IIa) after modified radical mastectomy (MRM). The patient underwent adjuvant chemotherapy and rehabilitation for her neurological condition. She regained functional independence with resolution of her physical impairment 3 months post-operation and remained disease-free since.

### Case presentation 2

**Fig. 2:**
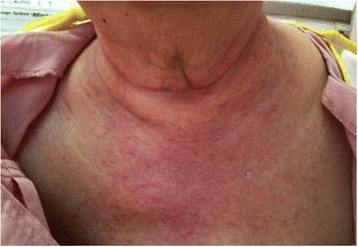
Erythematous rash typical of dermatomyositis on the patient’s neck and shoulders in a classical shawl distribution. Skin punch biopsy site on the left chest wall

**Fig. 3:**
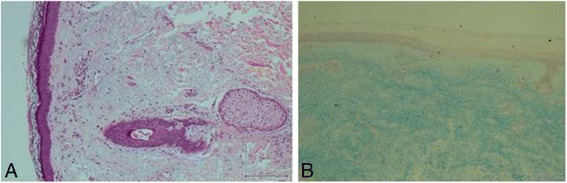
**a** Haematoxylin-eosin stain of skin biopsy showing perivascular and interface lymphocytes and dermis edema and mucin that are characteristic of dermatomyositis. (×100 magnification). **b** Alcian Blue stain of skin of the skin biopsy highlighting the increased dermal mucin in the superficial dermis that is characteristic of dermatomyositis. (×40 magnification)

A 69-year-old woman experienced 3 months of atypical chest pain, erythematous rash over her face and sun-exposed areas in a classical shawl distribution (Fig. 2), arthralgia, and proximal upper extremity muscle fatigue and weakness in a symmetrical distribution. She was admitted into cardiology by the emergency department. However, clinical impression of inpatient dermatology and rheumatology consults was dermatomyositis (DM). Creatine kinase 1409 U/L and aldolase 7.6 U/L were elevated, but anti-nuclear antibodies were negative. Electromyography showed myopathic changes, muscle biopsy displayed diffuse expression of MHC class I antigen on immunostaining supportive of underlying inflammatory myopathy, and skin punch biopsy was consistent with DM (Fig. 3).

Examination for an occult malignancy found a right enlarged axillary lymph node. Breast imaging found two irregular marginated nodules in the inner right breast with malignant features. Breast biopsy confirmed grade 3 IDC with negative oestrogen and progesterone receptor status but positive HER-2 receptor status. The cancer was staged as IIIc T_2_N_3_M_0_ after right MRM with a tumour size of 45 mm and 20 positive lymph nodes. Adjuvant chemotherapy, radiotherapy, Herceptin and oral prednisolone were administered. The dermal lesions, arthralgia and myasthenia completely regressed within 4 months post-operation, and the patient remained disease-free for 21 months. The DM rash, however, reappeared 21 months post operation, and a computed tomography scan performed confirmed disease relapse with radiological findings characteristic of hepatic and pulmonary metastasis.

### Discussion

The etiological relationship between cerebellar degeneration and malignancy was first detailed by Brain et al. in 1951 [4]. Graus et al. in 2004 designed a diagnostic criterion of a neurological syndrome as paraneoplastic based on the (1) presence or absence of cancer, the (2) definitions of classical syndrome and (3) well-characterized onconeural antibody [5]. PCD is mediated by onconeural antibodies (ONAs) produced against tumour antigens. Anti-Yo antibody is the most common ONA associated with PCD followed by anti-Hu, anti-Tr and anti-Ri [6, 7]. Cdr2, also known as Purkinje neuronal protein, is expressed on cells within the cerebellum and is similar to the tumour antigen that is expressed in breast and ovarian tumours for anti-Yo antibody [8]. Cross-reaction between the ONAs and normal proteins occur, resulting in abnormal immune-mediated responses that cause cerebellar injury and neuronal dysfunction. Animal models suggest that cdr2 downregulate c-Myc proto-oncogenes in the neuronal cytoplasm. ONAs have been demonstrated to disrupt cdr2-c-Myc interactions, potentially leading to increased c-Myc activity and eventual apoptosis of the Purkinje cells [9]. Recent literature suggests that while ONAs play an initial pathogenic role in PCD, and cytotoxic T-lymphocytes also contribute to the neuronal degeneration [10]. Cerebellar cognitive affective syndrome, in which slight memory and cognitive deficits and affective symptoms are displayed, may occur in 20 % [8]. Thorough investigation including CSF examination and serum tumour markers should be performed in a patient with PCD. CSF may have raised protein and IgG, predominant lymphocytic pleocytosis and positive oligoclonal banding as in our patient [11]. With disease progression, MRI brain scan may show generalized cerebellar atrophy [12, 13].

Treatment is aimed at the eradication of the malignancy with supportive rehabilitation of the cerebellar abnormality. No randomized control trials exist to establish a treatment protocol but attempts to treat the neurological dysfunction with chemotherapy, immunoglobulins, plasmapheresis and/or immunosuppression have yielded poor response [14]. While tumour prognosis and survival in negative anti-Yo antibody PCD patients is similar to those with normal breast cancer, anti-Yo antibody positive patients are reported to have a more aggressive cerebellar disease course with more than 90 % eventually requiring an assistive device for ambulation and significantly poorer overall survival (median 100 months) [11, 14]. Our patient highlights the better prognosis in negative anti-Yo antibody PCD.

DM is an autoimmune inflammatory myopathy with characteristic cutaneous findings that include pathognomonic heliotrope rash, Gottron’s papules or a rash eruption in a photosensitive distribution [15]. Proximal muscle fatigue or weakness may occur. In 1975, Bohan and Peter presented the criteria to aid diagnosis, and our patient met the definite criteria (Table 1) [16]. The association between DM and malignancy is well-established and reported in 15–30 % of adult-onset DM with increased frequency with age. Ovarian and lung tumours are the most commonly linked in female and males, respectively. Paraneoplastic DM is not associated with poorer prognosis and commonly demonstrates a parallel regression of the dermal lesions and muscular symptoms when the cancer is in remission and return with malignancy recurrence as highlighted by our patient. Hence, clinical follow up for these patients should include observation of recurrent cutaneous signs or myopathy. However, breast carcinoma may sometimes be associated with amyopathic DM which is characterized by the absence of myopathy. It is suggested that amyopathic DM is not responsive either to steroid treatment and does not regress despite eradication of malignancy.

**Table 1:** Bohan and Peter’s criteria for diagnosis of dermatomyositis

Individual criteria	
1. Symmetric proximal muscle weakness	
2. Muscle biopsy evidence of myositis	
3. Increase in serum skeletal muscle enzymes	
4. Characteristic electromyogram pattern of myositis	
5. Typical rash of dermatomyositis	
Diagnostic criteria	
• Definite: 5 plus any three of 1 to 4	
• Probable: 5 plus any two of 1 to 4	
• Possible: 5 plus any one of 1 to 4	

## Conclusions

In conclusion, while tumour prognosis per se is similar to patients who present in the normal manner, early tumour eradication slows the autoimmune response, allowing a chance for paraneoplastic symptom remission or stabilization. Our cases demonstrate the vitality to maintain a high level of clinical vigilance for paraneoplastia and assessment for malignancy upon initial diagnosis to prevent delayed management and irreversible damage, neurological or otherwise.

## Consent

Written informed consent was obtained from the patient for publication of this case report and any accompanying images. A copy of the written consent is available for review by the Editor-in-Chief of this journal.

## Abbreviations

CSF: cerebrospinal fluid
CT: computed tomography
DM: dermatomyositis
IDC: invasive ductal carcinoma
Ig: immunoglobulin
MRI: magnetic resonance imaging
MRM: modified radical mastectomy
ONA: onconeural antibodies
PCD: paraneoplastic cerebellar degeneration
